# A framework for unit-level biodiversity credit integrity and market-level governance

**DOI:** 10.1038/s44185-026-00161-6

**Published:** 2026-10-02

**Authors:** Lara Marie Pelka, Megan Meacham, Hassan Aftab Sheikh

**Affiliations:** 1 Department of Sustainable Development, Environmental Science and Engineering, School of Architecture and the Built Environment, Kungliga Tekniska högskolan, 100 44 Stockholm, Sweden; 2 Stockholm Resilience Centre, Stockholm University, 106 91 Stockholm, Sweden; 3 Smith School of Enterprise and the Environment, University of Oxford, Oxford, OX1 3QY UK

**Keywords:** Ecology, Ecology, Environmental sciences, Environmental social sciences

## Abstract

Biodiversity credits (BCs) are one innovative financing scheme that the Global Biodiversity Framework proposes to mobilise private capital and narrow the biodiversity financing gap. BCs and the voluntary biodiversity markets (VBMs) around them are nascent, offering an opportunity to learn from shortcomings of the adjacent voluntary carbon markets and design them for both ecological and social integrity from the outset. This paper examines the requirements for the effective functioning of BCs and VBMs at the unit and market levels. We propose a requirements framework based on a horizon scan and semi-structured interviews with market participants. Key five unit-level criteria, comprising four environmental integrity criteria (measurement and monitoring, durability, additionality and leakage minimisation) and one social integrity criterion (environmental and social safeguards), are proposed. At the market level, three components underpin functioning VBMs: clearly defined, fairly allocated and easily transferable rights. Building on lessons from carbon markets, we derived mitigation measures to guide the development of an improved market architecture.

## Introduction

Biodiversity, fundamental to life on Earth, keeps declining at an unprecedented rate^1,2^ due to land and sea use change, direct resource exploitation, climate change, pollution and invasive species^3^. Individual species are threatened with extinction^4^ as are ecosystems, both terrestrial and marine^3^. This biodiversity crisis directly affects societal and economic well-being^5^. Humans are dependent on biodiversity for food, medicine, energy, clean air and water and protection from natural disasters, but also for recreational purposes and cultural inspiration^6^. More than half of the world’s GDP is dependent on nature^7^.

World institutions, such as the United Nations (UN), have recognised the criticality of the current biodiversity crisis and have put in place policies and frameworks to address it. One cornerstone framework is the Kunming-Montreal Global Biodiversity Framework (GBF), adopted in 2022 by 196 countries, that aims to “halt and reverse biodiversity loss” by 2030^6^. The GBF sets out 23 targets, including direct protection of terrestrial and marine ecosystems and the inclusion of indigenous peoples and local communities (IPLCs); however, several targets also address the financial means needed for these efforts (ibid.). Deutz et al.^1^ estimated the annual funding gap for biodiversity to be around $700 billion. Until recently, primary funding sources for biodiversity conservation and restoration efforts have been public funding and philanthropic sources^8^. While public funding has seen a positive upwards trend^9^, overall budgets remain too low to efficiently address biodiversity loss^10,11^. Hence, there is a need for more private funding for biodiversity. Target 19 puts emphasis on the role of private funding for biodiversity, calling for the stimulation of “innovative schemes such as payment for ecosystem services [PES], green bonds, biodiversity offsets and credits”^6^.

The focus of this paper is biodiversity credits (BCs) and the markets around them. To understand the need for BCs, it is important to appreciate what differentiates them from the other “innovative schemes,” such as PES, green bonds and biodiversity offsets (BOs), that have existed for some time^12^.

PES are bilateral agreements paying landowners to maintain certain ecosystem services (ES)^13,14^. PES are performance-based biodiversity financing mechanisms^15^, but they have faced multiple issues as a conservation strategy^16^, including human-centricity and neglect of ES that are critical to nonhumans; mismatch between conservation and ES optimisation via exotic species; and maximisation of single-service provision leading to increased ecological brittleness (ibid.).

Green bonds are debt instruments whose proceeds are dedicated to financing projects with positive environmental impacts^17,18^. As conventional bonds, green bonds are linked to a direct financial return (ibid.). They can be used for biodiversity conservation projects, both for impacts that can be easily commodified (e.g. more sustainably produced raw materials) and for impacts more difficult to commodify, previously having received funds from philanthropic sources (e.g. increasing a species population)^19^. While green bonds have seen large uptake ever since their first introduction in 2007^17^, main challenges with green bonds include the need for a financial return, which not all projects can offer, and the mismatch between tenures and actual required time horizons to achieve long-lasting biodiversity impact^19^.

Biodiversity offsets (BOs) are typically used in compliance contexts to compensate for residual harm from development activities. Conceptually, offset credits can be understood as a subset of BCs, where the demand driver is compensatory (i.e., offsetting), rather than voluntary contribution^20^. BOs are purchased in compliance with biodiversity markets (e.g. in the UK), or via mandatory BO schemes (e.g. in Germany)^21^. One major weakness of this financial instrument is that, except for the Biodiversity Net Gain scheme in England (https://www.gov.uk/guidance/understanding-biodiversity-net-gain), mandatory BOs usually aim to compensate for harm done elsewhere, not to improve biodiversity’s state.

BCs, sometimes also referred to as Nature Credit, BioCredit, or Biodiversity Unit^22^, have started to emerge more recently. BCs are a tradeable unit representing a measured and evidence-based positive biodiversity outcome^23^. For BCs to be considered “high-integrity,” they must meet specific performance criteria over time, e.g. to be “durable and additional to what would have otherwise occurred”^24^. In that sense, BCs support biodiversity targets that are SMART (Specific, Measurable, Achievable, Relevant, Time-limited). This is ideal as such biodiversity targets allow for actual tracking of progress, fostering the targets’ achievement^25^.

We define the SMART criteria in this paper as: (1) Specific: The *unit of positive biodiversity outcome* (target) refers to what a BC actually represents based on the underlying project type^26^, e.g. avoidance (reduction in biodiversity threats), maintenance (prevention of anticipated biodiversity decline), or restoration (biodiversity improvement) projects^24^. The biodiversity outcome is measured as the delta between the scenario with and without project activities over a specified time horizon (ibid.), making the biodiversity target specific. (2) Measurable: By definition, BCs’ biodiversity outcomes are *measured* (hence measurable) and *evidence-based*, because biodiversity is quantified based on selected indicators and any change in biodiversity is monitored over time^26^. (3) Achievable: As long as BCs rely on ex-post issuance, biodiversity outcomes’ achievability is inherently incentivised as credits can only be issued once the outcome has been demonstrated^26^. (4) Relevant: The relevance of BCs’ biodiversity outcomes is anchored in the requirement of BCs to be *additional* and *durable*^26^. (5) Time-limited: A specified project time horizon sets out a clear limit by which the targeted biodiversity outcome should be achieved.

Beyond PES, BCs aim to represent biodiversity more holistically through the selection of multiple relevant indicators. Unlike PES, they are not limited to a single ecosystem service (ES) or the desirability of intact ES for humans. BCs also seek higher levels of standardisation, tradability and traceability than PES^22^. Compared to green bonds, BCs can target longer project time horizons and emphasise the durability of desired outcomes. In contrast to mandatory BOs, BCs are designed not only to maintain but also to improve biodiversity, offering companies an opportunity to contribute to conservation beyond regulatory requirements, particularly those that would otherwise not have engaged in such efforts^26,27^ (https://www.biodiversitycreditalliance.org/faqs/).

**Fig. 1:**
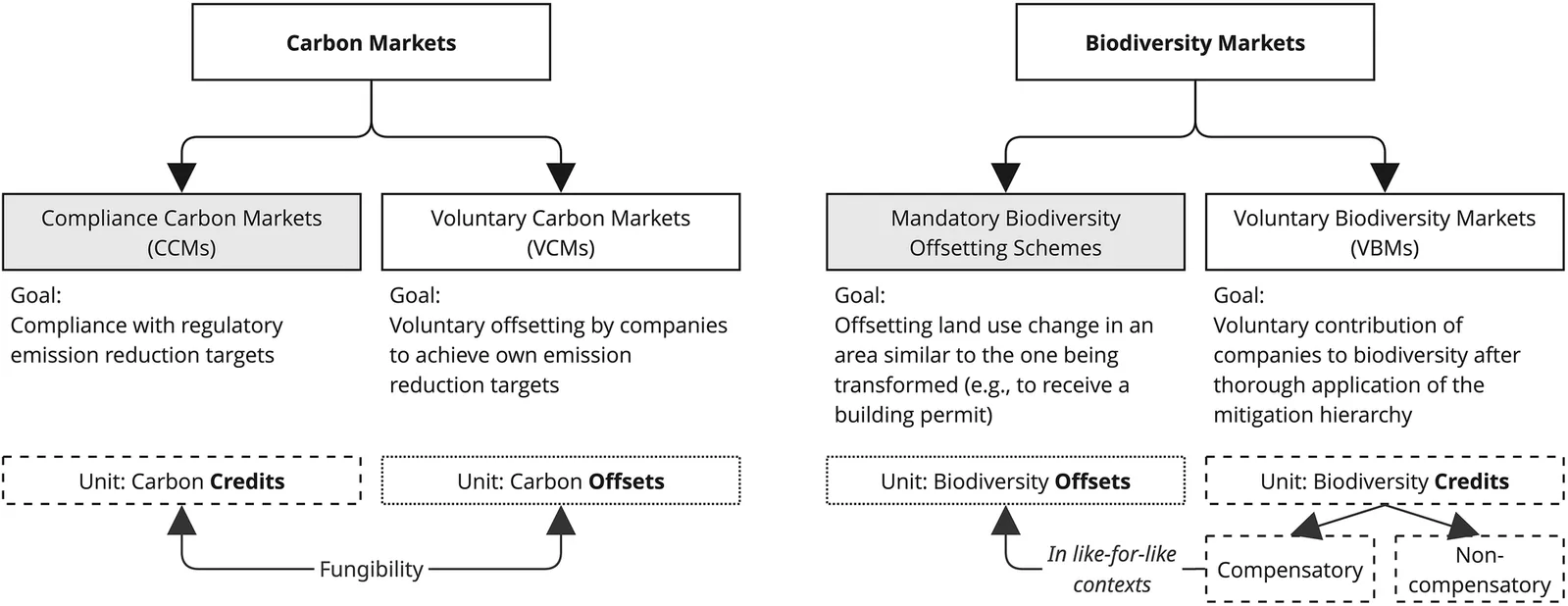
Overview of carbon and biodiversity markets. Schematic comparison of compliance and voluntary carbon and biodiversity markets, including compliance carbon markets (CCMs), voluntary carbon markets (VCMs), biodiversity offsets (BOs) and voluntary biodiversity markets (VBMs). The figure illustrates how biodiversity credits (BCs) and the VBMs conceptually build on carbon credit mechanisms while differing in fungibility, spatial specificity and permitted offsetting approaches. It further distinguishes compensatory and non-compensatory biodiversity credit use cases and situates BCs relative to other biodiversity finance instruments, including payments for ecosystem services and green bonds.

The mechanism behind BCs imitates the concept of carbon credits (CCs), or carbon offsets, and voluntary biodiversity markets (VBMs), the markets via which BCs are traded, imitate voluntary carbon markets (VCMs), as Fig. 1. illustrates. BCs and CCs are both market-based instruments that aim to contribute to the improvement of biodiversity and the mitigation of the climate crisis, respectively. Both attempt to package biophysical processes/ecosystem services into a measurable, tradeable unit based on a project^24,28,29^. In the case of CCs or carbon offsets, units are based on CO_2_ equivalents (https://www.unep.org/topics/climate-action/climate-finance/carbon-markets), in the case of BOs/BCs, units are usually based on multiple indicators aiming to represent biodiversity’s complexity^26,27^.

As Fig. 1 shows, carbon and biodiversity markets can be divided into compliance markets, which have existed for decades, and voluntary markets, which have emerged more recently. VCMs and VBMs primarily mirror carbon compliance markets (CCMs), which are regulated markets that require companies to offset harm done elsewhere^27,28,30,31^, and allow for the trading of units within and across national borders, e.g. the European Union (EU) Emissions Trading System (https://climate.ec.europa.eu/eu-action/eu-emissions-trading-system-eu-ets/what-eu-ets_en). Mandatory BO schemes, on the other hand, represent purchasing programmes rather than standard markets and usually exist only on a national scale^21^. Voluntary markets, like VCMs and VBMs, aim at mobilising capital mainly from companies that want to show commitment beyond regulatory requirements^28^. The main difference between VBMs and VCMs is the degree to which offsetting is allowed. VCMs operate under the equivalence principle and build on voluntary offsetting by market participants, which is possible because CCs/carbon offsets are fungible, as it does not matter where emissions are reduced as long as they are^30^. To streamline terminology, units traded in either CCMs or VCMs are referred to as CC going forward, which is possible because of the fungibility mentioned. Literature uses both terms interchangeably. Biodiversity, on the other hand, is highly location-specific^27^, which implies that offsetting in VBMs (compensatory use case) can only be permitted in like-for-like contexts. The other use case in which BCs are expected to play a role is of a non-compensatory nature: BCs are supposed to come into play after the thorough application of the entire mitigation hierarchy—after the avoidance of impacts on biodiversity where possible, minimisation, restoration and offsetting of any unavoidable impacts^32^. In this use case, BCs are intended to mobilise capital from actors who want to make voluntary contributions to biodiversity.

Despite the differences, it becomes evident that BCs and CCs stem from the same school of thought and are similar enough to transfer learnings from the carbon to the biodiversity side. Considering the criticism and negative reputation associated with VCMs emphasised in almost all BC-related literature (cf^27,33^) and the fact that BCs and VBMs are only now emerging, it is essential to draw lessons from the carbon side and apply these insights to BCs while they can still be shaped. Otherwise, there is a risk of low-quality credits “weak demand, poor supply, slow uptake, high costs, lack of outcomes and potentially market failure”^34^.

Recent work has begun to establish the scientific foundations for high-integrity nature markets. Zu Ermgassen et al.^35^ synthesise decades of research to propose five rules for scientifically credible nature markets, scoring existing markets against these rules. Kim et al.^36^ provide an empirical assessment of how existing BC standards handle social performance criteria, including FPIC protocols. Wunder et al.^22^ offer a comprehensive overview of the current state and pitfalls of BCs. This growing body of work provides strong theoretical grounding for what high-integrity BCs should achieve. However, three complementary contributions remain underexplored: (1) independent triangulation of these principles through expert interviews with current market participants, surfacing implementation challenges, contested areas and feasibility constraints not captured in desk-based reviews; (2) an integrated market-architecture analysis grounded in economic market theory^49^, extending the unit-level focus of existing reviews to include market-level governance design; and (3) a direct mapping of lessons from VCMs onto the BC context. This paper addresses this research gap by providing key criteria and components that need to be satisfied for BCs to function effectively at the unit and market levels. We systematically transfer learnings from the carbon to the biodiversity side and propose an improved VBM architecture compared to one that would exactly mirror a VCM-type architecture. Our research is carried out through a horizon scan and semi-structured interviews. We aim to contribute to the best possible setup of VBMs to achieve both ecological and social integrity at scale. We further follow recent calls by Karolyi and Tobin-de La Puente^37^ for more research on biodiversity finance, and by Flammer et al.^8^, particularly concerning private capital in biodiversity.

## Results

**Fig. 2:**
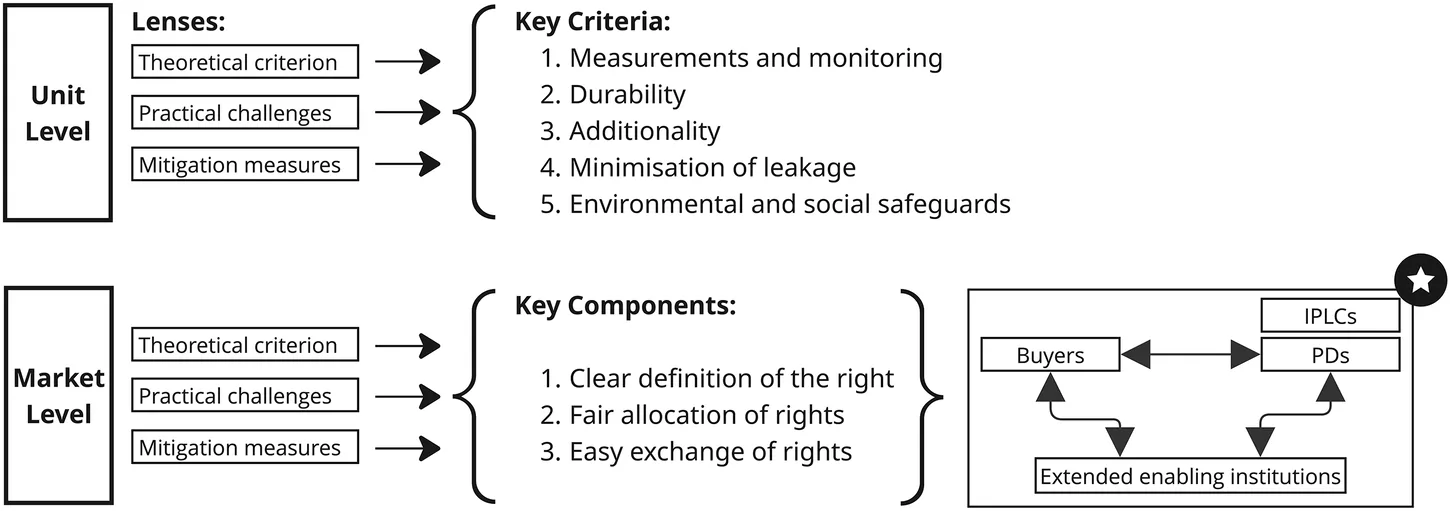
BC requirements framework. Framework summarising the requirements for effectively functioning biodiversity credits (BCs) and voluntary biodiversity markets (VBMs). The framework separates unit-level criteria, including measurement and monitoring, durability, additionality, leakage minimisation and environmental and social safeguards, from market-level governance components related to the definition, allocation and exchange of rights. Each component is evaluated through theoretical definition, practical challenges and mitigation measures. Abbreviations: BC biodiversity credit, PD project developers, VBM voluntary biodiversity market, IPLCs indigenous peoples and local communities.

We identified the requirements for effectively functioning BCs and VBMs via a horizon scan and semi-structured interviews. To organise and present the findings, we created the *BC Requirements Framework* (cf. Fig. 2), which serves as the guiding structure for the results. To structure the analysis, we distinguish between requirements that apply to individual BC units and requirements that apply to the market as a whole. Unit-level requirements concern whether a credit credibly represents a measurable, durable, additional and socially legitimate biodiversity outcome. Market-level requirements concern the governance, institutional and infrastructural conditions needed for BCs to be issued, traded, claimed and overseen in a credible market. This distinction provides the analytical structure for the BC requirements framework presented below.

### Unit level

At the unit level, we distinguish between environmental integrity and social integrity criteria that underpin effective BC function, drawn from the BC definition^24^ and core BC and CC literature (e.g. ^12,22,26,27^). Four of the five criteria: measurement and monitoring, durability, additionality and minimisation of leakage relate to environmental integrity, e.g. whether a BC represents a real, measurable change in ecological outcomes. The fifth criterion, environmental and social safeguards, relates to social integrity, addressing the justice, rights and distributional implications of BC generation and exchange. The first three mirror standard requirements for carbon credits^28^, while the last two come from more recent BC research^26,27^.

Although these criteria should, in theory, ensure functional BCs, practical experience with CCs shows that each faces implementation challenges^26,33^. Hence, to prevent the ineffective functioning of BCs, it is critical to understand what could hinder the criteria from being met. Therefore, we examined each criterion through three lenses: theoretical definition, practical challenges and mitigation measures, summarised in Table 1. The theoretical definition expands on the specifics of the criterion. The practical challenges describe barriers that this criterion might face inherently or is likely to face given BCs’ resemblance with CCs. Mitigation measures propose solutions to mitigate the challenges and/or ease the barriers.

**Table 1:** Summary of the challenges and mitigation measures around the five key criteria for performance and integrity

Criterion	Challenges	Mitigation measures
Measurement and monitoring	Non-holistic representation of biodiversity and its value	*BC-specific*
	Combining metrics can hide trade-offs between unrelated measures	*BC-specific*
	Natural fluctuations can lead to commodifying noise	Application of long-time horizons and establishment of control sites
	Costly implementation of complex monitoring techniques	Affordability increases and higher project credibility might outweigh costs
	Overly large monitoring intervals	Different intervals for different indicators
	Data migration to remote actors	Inclusion of data protection and access within benefit-sharing mechanisms
Durability	Natural and anthropogenic forces undermining permanence	Application of long time horizons, establishment of buffers and set-up of adjustment plans
	Preference for short-term contracts	Coupling of BC use case and project duration/specific milestones
Additionality	Artificial financial additionality and uncertainty around counterfactuals	Establishment of control sites, increase of buffers and promotion of ex-post issuance
Minimisation of leakage	Difficulty in measuring both direct and indirect leakage	Risk analyses & control data
Environmental and social safeguards	Harming biodiversity for carbon removal	Everything depends on the selection of indicators
	Narrow project focus	Awareness and active scope broadening
	IPLC discrimination and exclusion	Guarantee of IPLCs’ rights before the transaction

BCs quantify biodiversity gains through chosen metrics and monitoring techniques, yet no common standard exists (Interview #4, Sustainability Advisor and Sustainability Specialist; #6, Biodiversity Markets Expert). BCs aim to capture biodiversity, the diversity within and between species across ecosystems^3^. Unlike the single CO₂ metric in CCs, biodiversity measurement is inherently approximate and must be ecosystem-specific and science-based^22,27,38^. There are two distinct measurement approaches for BCs:

1. Binary indicators indicate whether a condition is met, e.g. species presence/absence^26,39^.
2. Basket of metrics combines metrics of structure, function, composition and area into a single value, typically normalised (relying on a “distance to target” approach), weighted (by assigning relative importance), or aggregated (e.g. by taking the average)^24,26^.

Multiple tools support biodiversity measurement for baseline setting and ongoing monitoring. Two broad monitoring types provide evidence of change (or maintenance): ex novo approaches that collect new data, e.g. through field-based surveys, Light Detection and Ranging technology, camera traps, passive acoustic monitoring, or environmental DNA^22,33^, and desktop approaches that process existing generic or site-specific geodata^22^.

Measurement and monitoring face several challenges. First, metrics privilege what is quantifiable, omitting dimensions that are “technically challenging or conceptually impossible to quantify”^26^, e.g. species interactions or nature’s cultural value, especially to IPLCs. Particularly, binary indicators oversimplify biodiversity’s complexity and are not representative^26,39^. Second, composite indices may mask trade-offs among unrelated measures (e.g. population size vs. species richness)^26^. The “distance to target” approach is also problematic, as ideal targets are often unknown, specifically in the face of rapid climate change, making this approach vulnerable to manipulation^26,39^. Both challenges are highly biodiversity-specific, and there are no direct lessons learned from CCs, which only consider the one-dimensional metric of CO_2_(e), as Table 1. shows. A further measurement challenge is that most metrics naturally fluctuate through time, e.g. variation in weather patterns such as El Niño Southern Oscillation can influence species population dynamics even in well-protected areas^33^. Attributing change in an ecosystem’s state to a project rather than natural dynamics risks “commodifying noise”^26^. Such risk can be mitigated by ensuring long enough time horizons to avoid rewarding or penalising random fluctuations. Control sites can also provide additional insight and minimise the risk of overinterpretation^33^.

On the monitoring side, capability and affordability have improved for multi-component, local-impact assessment^33,40^, though in practice interviewees emphasised that correct biodiversity measurement and monitoring is still complex and expensive, factoring into the BC price (e.g. Interview #3 and Impact Director). The most cost-effective monitoring approach is expected to combine field surveys with new ex-novo and desktop technologies; space-based measurement and monitoring remains debated^22^. The integrity of BC schemes depends on appropriate monitoring frequency: long or overly flexible intervals increase uncertainty and gaming risk; in basket-of-metrics approaches, intervals and methods should be tailored to each indicator to balance accuracy and resource intensity^22^. Lastly, remote monitoring approaches pose the risk of biodiversity data being displaced from local biodiversity stewards to remote actors, threatening equity and data justice^22^. While there is generally a stronger focus on the inclusion and participation of IPLCs in BC projects compared to the early days of CCs^22^, biodiversity data protection and access might play a particular role in negotiating benefit-sharing mechanisms.

Durability, or permanence, ensures that biodiversity outcomes persist, ideally for at least 20 years^24^. In compensatory use cases, generated benefits must match the duration of the impacts they offset^22^. Non-compensatory uses may deviate, but all BCs should still aim for long-lasting ecological benefits^22^. As with other outcome-based criteria, durability can only be fully assessed ex post and therefore represents a performance criterion more than a defining feature of a BC.

While no direct evidence yet shows that BCs face the same durability issues as CCs, lessons from CCs, PES and BOs, which have been criticised for insufficient permanence, suggest similar risks^22,26^. CC projects have struggled with natural and human pressures, e.g. wildfires, pests, invasive species, droughts, clear-cutting and contract breaches^26,28,30^. To address these, literature highlights buffers, long time horizons and adjustment plans: BCs should maintain buffer pools to cover unforeseen losses, avoiding the underestimation seen in CC schemes (ibid.).

While many projects aim for 20-year horizons^26,41^, scholars argue for 30–50 years or longer^33^, supported by long-term management plans including perpetual financing mechanisms and formal land-use restrictions on restored/protected areas to help improve the durability of conservation and restoration gains^22^. Standards like the Swedish Biocredit Alliance^41^ require plans for unforeseen events or additional loss, ideally verified by validation and verification bodies (VVBs) and reported to and approved by the buyers.

Another durability challenge observed in BO schemes is that land managers favour shorter contracts to avoid being locked into financially suboptimal forms of land management^22^ particularly under conditions of market uncertainty where future revenue streams remain unclear. Such unpredictability could be mitigated through staged investments that transfer capital incrementally from buyer to project developer (PDs) based on the achievement of project milestones^30^. Such payment structures may provide a degree of operational predictability by establishing recurring evaluation and payment cycles, particularly relevant to actors with long-term planning horizons. Additionally, such performance-based payments also align ecological and economic incentives on the supply side: One PD mentioned in an interview that such incremental payment, e.g. on a yearly basis, incentivizes them to perform well in terms of biodiversity improvement, as otherwise payments might stop the year after (Interview #2, Fund Director). While such mechanisms do not resolve uncertainty regarding long-term revenues and prevent more profitable opportunities from arising in the future through alternative land use, they may support ongoing project planning and financing by structuring financial flows into shorter, more predictable intervals. This might also accommodate buyers with generally shorter investment horizons who would otherwise step away from participating in VBMs, as investment cycles do not align with project timelines (e.g. Interview #1, Sustainability and Biodiversity Specialist and Environment Specialist).

Additionality is a core performance criterion for BCs referring to whether a project delivers measurable environmental benefits beyond what would occur under a baseline or business-as-usual scenario, and that these outcomes are attributable to the project (e.g. biodiversity conservation or restoration or CO_2_ reduction)^24,26–28,30^. In contrast to a purely definitional attribute, additionality can only be fully assessed over time, requiring post-implementation monitoring and verification.

The literature distinguishes several forms of additionality. BC literature has primarily emphasised *financial additionality*—projects that would not proceed without credit revenues^24,26^, while CC frameworks also emphasise *regulatory additionality*, excluding projects mandated by law from credit generation^28^. Together, these highlight that additionality is not a binary property but a multi-dimensional integrity condition that must be demonstrated empirically.

A credible baseline (counterfactual scenario) is central to demonstrating additionality^22,24,33^. Baselines describe expected ecological conditions in the absence of the project, typically referencing pre-project conditions and projected trends. Restoration projects should show measurable improvement relative to this baseline, while conservation projects must demonstrate no decline or a lowered pace of decline^26^. Baselines may be adjusted to reflect expected biodiversity loss in the project’s absence, and they may allow limited deviation before no more credits are issued^26^. However, because baselines represent counterfactual futures, they are inherently uncertain and cannot be directly observed.

Experience from carbon markets shows that baseline construction is not merely a technical challenge but a core source of integrity risk. A central failure mechanism in the voluntary carbon market (VCM) has been baseline inflation, where project developers systematically overstate the severity of the “without-project” scenario to maximise credit issuance, producing so-called *“hot air”* credits.

Empirical evidence from the carbon literature provides a strong cautionary signal for biodiversity markets. Studies (e.g. ^42,43^) and investigative analyses (e.g. https://www.theguardian.com/environment/2023/jan/18/revealed-forest-carbon-offsets-biggest-provider-worthless-verra-aoe) have shown that a substantial share of rainforest carbon offsets were effectively non-additional due to overestimated deforestation baselines. Similarly, cookstove projects have been found to systematically over-credit due to unrealistic counterfactual assumptions. These outcomes are not always the result of deliberate manipulation but often emerge from methodological flexibility, outdated data and entrenched sector practices that gradually normalise optimistic baselines.

Beyond baseline construction, BCs face risks of adverse selection, where projects are located in areas with low actual threat levels. In these scenarios, developers select regions, landowners, or ecosystems where conservation or restoration would likely have occurred anyway, allowing projects to capture credit revenues without generating real ecological gains. This dynamic further undermines additionality and is particularly acute for avoided-loss (conservation) projects, where counterfactual decline is difficult to verify^22,26^.

CC experience shows that financial additionality was often overstated, with removal projects (e.g. direct air capture) seen as more credible than avoidance ones, as the required investment was easier to demonstrate^28^ (Interview #4, Sustainability Advisor and Sustainability Specialist). Similarly, BCs may demonstrate stronger additionality in technology-based restoration than in low-cost, nature-based efforts.

Establishing credible baselines is inherently uncertain, as with all counterfactuals^28^. CC projects often received criticism because they used a variety of methodologies to determine baselines, relied on outdated information, e.g. deforestation trends, preceding immediate project start^28^, and often overestimated future gains^33^—all of which misrepresent the counterfactual or otherwise undermine the credibility of additionality claims. Analyses show that about half of existing BC schemes are either action-based and do not measure additionality, or they lack clear methodologies to establish a baseline and determine additionality^22^.

Addressing these challenges requires shifting the additionality assessment from ex-ante assumptions to ex-post verification frameworks. Mitigation measures for additionality and baseline challenges include control sites, larger buffers and the promotion of ex-post issuance. Control sites, similar to the project site in terms of ecological integrity and/or threat profile^24^ and not self-selected^22^, help demonstrate counterfactuals, through many developers still use static rather than dynamic baselines^39^, echoing CC shortcomings^33^. Expanding buffer pools in place for potential impermanence can help mitigate additionality uncertainties^26^, provided they are set at sufficient levels, as learnings from CCs show. Lastly, BCs should be issued only after outcomes have been verified, ideally via satellite data, though virtually no BC scheme follows this recommendation yet^22^.

An alternative view on additionality presented by an interviewee argues that, given the urgency of the biodiversity crisis, the additionality question can be circumvented by accepting that any intact ecosystem is worth protecting via a project, and the measured status quo or changes in biodiversity over time simply affect what claims can be made about this unit (Interview #5, Project Manager).

Leakage describes the displacement of an impact elsewhere^44,45^. It should be avoided to the highest degree possible in BC as in CC projects, as it reduces or even negates project benefits^26,30^. Leakage can be direct (primary/local) referring to the direct displacement of harmful impacts, e.g. restoring one agricultural field, but moving production to a wild area nearby; or indirect (secondary/market), e.g. if land is redirected from crop production to a conservation project, crop supply decreases in that place, crop market prices are likely to rise, potentially incentivising farmers elsewhere in the world to expand their crop production instead^26,44,45^. The more price-elastic the goods, the greater the leakage risk^22^.

Leakage has posed significant challenges in VCMs and BO schemes, primarily because it is difficult to accurately measure, yet it can have significant negative effects on the broader landscape^12,26^ (Interview #7, Former Policy Advisor). Measuring direct leakage in BC projects would require monitoring biodiversity within the credit-generating area and surrounding land^26^, posing challenges of determining an appropriate spatial and temporal scale^33^, and potentially resulting in significant additional efforts^39^. Market leakage is almost impossible to reliably measure because of global-scale teleconnections^26,28^.

“There is no consensus on how leakage is best addressed” (p. 14)^22^, but options include risk analyses, e.g. identifying and mitigating potential leakage risk factors and putting controls in non-project areas in place^41^, and larger buffers to cover potential losses^26^. However, guidance on implementation remains limited.

Environmental and social safeguards constitute the social integrity dimension of BCs, complementing the biophysical integrity criteria discussed above. While earlier criteria focus on ecological outcomes, this principle addresses the justice, rights and distributional implications of credit generation.

GBF Target 19 calls for benefit-sharing mechanisms with environmental and social safeguards^6^. Both BC and CC projects should follow those safeguards, aiming to reduce and mitigate physical, social, economic and environmental risks^30^, thereby achieving “No Net Harm.” This requires avoiding harm to both ecosystems and local communities, supported by active and inclusive stakeholder management^28^. However, high-integrity approaches increasingly require moving beyond “No Net Harm” toward Rights-Based Approaches, ensuring that projects actively support equitable and just outcomes.

A central element of this is the rigorous implementation of Free, Prior and Informed Consent (FPIC), ensuring that IPLCs retain agency over their lands and are not displaced or marginalised by conservation interventions. While current standard setters, such as Verra^46^ and Plan Vivo^47^, emphasise the inclusion and compensation of IPLCs’ stewardship, evidence suggests that the depth and consistency of FPIC implementation vary across frameworks, and often remain stronger in principle than in practice. This highlights the need for verifiable enforcement of rights, not only procedural inclusion.

Benefit-sharing mechanisms could include minimum percentages for IPLCs or the establishment of price floors^30^. Further, Antonelli et al.^27^ emphasise that IPLCs should be ensured the right to manage their lands according to traditional practices, pointing toward a broader shift from benefit-sharing to co-development and collaborative governance, where IPLCs are central to project design and decision-making rather than passive recipients of benefits.

Experience from CC projects shows persistent weaknesses in safeguard implementation. Historically, environmental safeguards have often been underdeveloped in CC projects^48^, e.g. harming biodiversity for the sake of carbon removal^27,30^. For CCs, one common risk mitigation strategy against biodiversity loss has been to enhance CCs with biodiversity co-benefits and sell these enhanced credits at a premium^30^. By contrast, BCs already integrate multiple biodiversity indicators, but indicator selection strongly influences whether outcomes are truly positive. Further, CC projects have been criticised for having a narrow focus on forests, ignoring other important carbon sinks^27^. Many BC projects start falling into a similar trap, primarily targeting terrestrial ecosystems (https://airtable.com/appXHR0Nau8HqfPa8/shrt5JZqmLMrQgdfF/tblTM31mqfzbGGBtR/viwLkSPMhe3K76baF). Broadening project scopes to also include marine and other ecosystems can mitigate this risk.

Social safeguards have also shown mixed successes in past CC and BO projects^22,27^, with instances of IPLCs being displaced for a project’s sake and/or excluded from benefits^12,26,28,30^. Given their nature, BC projects face similar risks, though current initiatives place stronger emphasis on safeguards compared to the early stages of CCs^22^. PDs should certify to the buyer that IPLC rights are protected before any transaction is undertaken, including fair compensation of IPLCs for active stewardship and legally backed benefit-sharing^27^. Current standard setters, such as Verra^46^ or Plan Vivo^47^, emphasise the active inclusion and compensation of IPLCs’ nature stewardship in their frameworks, though the credibility of promises depends on future project implementation^22^.

### Market level

Markets are complex webs of institutions and social arrangements designed to enable transactions at minimal cost^49^. Properly functioning markets depend on three elements: clearly defined rights over the traded good or service; an initial allocation of rights that balances competing principles of allocation (e.g. prior use or efficiency); and smooth exchange of rights supporting easy transferability and enforcement to keep transaction costs low^49^. These are particularly relevant for emerging markets such as VBMs. We therefore analyse (i) the “web of institutions and social arrangements” of current VBMs, here termed market participants, and (ii) the three components, through lenses of theoretical definition, challenges informed by VCM shortcomings, and mitigation measures proposed for VBMs or adapted from VCMs.

**Fig. 3:**
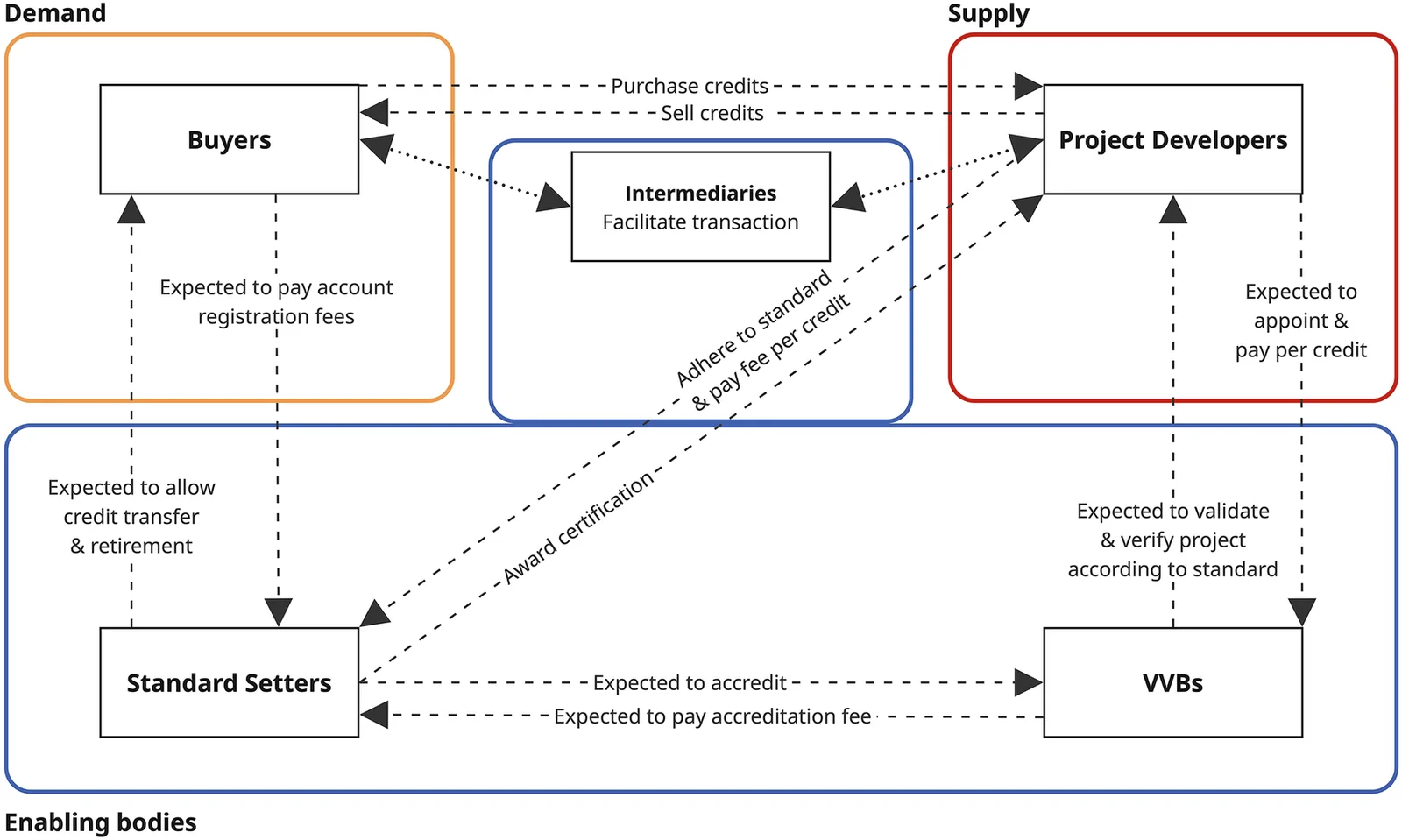
Schematic overview of market participants in a VCM-type architecture. Adapted from Battocletti et al.^28^. The figure illustrates a voluntary biodiversity market (VBM) architecture modelled after voluntary carbon markets (VCMs), showing interactions between buyers, project developers (PDs), standard setters, validation and verification bodies (VVBs), registries, brokers, marketplaces and financial institutions. It highlights how biodiversity credits (BCs) are developed, validated, issued, transferred and retired within the market system. Abbreviations: VVB validation and verification body.

Our analysis shows that VBMs remain in an early, highly fragmented stage (e.g. Interview #1, Sustainability and Biodiversity Specialist and Environment Specialist; #3, Impact Director). Emerging participants can be grouped into three categories: demand (buyers), supply (project developers (PDs)) and enabling institutions (standard setters, VVBs, intermediaries). Figure 3 illustrates a VBM architecture mirroring that of VCMs, with roles and interactions between participants. PDs design projects underlying BCs using methodologies issued by standard setters, who in turn rely on VVBs to audit quality requirements, ensuring double surveillance. After validation and verification, BCs are issued and sold directly to buyers or via intermediaries such as brokers or marketplaces. Buyers typically pay a fee to standard setters to transfer or retire credits from registries^28^. Focusing primarily on Europe, interviewees described having to occupy multiple roles simultaneously because of limited institutional market infrastructure (e.g. Interview #1, Sustainability and Biodiversity Specialist and Environment Specialist; #3, Impact Director).

“*We try to really be […] just a methodology developer, but we realise that we need to be in several different places at the same time, just to be able to help out in creating the markets.” (Interview #3, Impact Director)*

The supply side appears most advanced: numerous heterogeneous PDs, both for-profit and non-profit, operate under different BC schemes (https://airtable.com/appXHR0Nau8HqfPa8/shrt5JZqmLMrQgdfF/tblTM31mqfzbGGBtR/viwLkSPMhe3K76baF—accessed version: January 2025). Enabling institutions are slightly less mature, but emerging: As of 2025, several standards exist^24,46,47^, though the market is not yet consolidated. Like carbon markets, it is expected to concentrate around a few credible gatekeepers as larger standards gain trust through scale^28^.

While no officially accredited VVBs yet exist, transactions have occurred following ex-ante validation and ex-post verification (Interview #3, Impact Director). Intermediaries include market intelligence platforms (e.g. BloomLabs), third-party brokers (e.g. Treebula), and financial institutions building internal processes to connect PDs and buyers (Interview #1, Sustainability and Biodiversity Specialist and Environment Specialist). Some banks have even started securing BC supply in anticipation of future resale (Interview #3, Impact Director). For example, Swedbank has a pilot project which involves the acquisition of BCs from Orsa Besparingsskog (a community-owned forest organisation in Orsa, Sweden, that manages local forest land https://www.swedbank.com/sustainability/environment/biodiversity.html).

The demand side is the least mature. Most European transactions are experimental, with supply vastly exceeding demand at the moment (Interviews #2, Fund Director; #3, Impact Director; #5, Project Manager). As in any new market, VBMs likely require pioneer buyers in the beginning. While some argue that sustained scaling will eventually require participants to generate profits to attract private capital, this is only one potential pathway for market development^50^. Alternatively, BCs may first scale within existing philanthropic and public conservation. In this view, BCs serve to standardise, measure and improve the transparency of existing efforts without necessarily requiring new, profit-driven investment models. Whether profit is a necessary driver for scale or whether BCs will primarily serve as a rigorous monitoring framework for nonprofit conservation remains an open question.

In summary, two insights arise: First, VBMs are likely to mirror VCMs to a large extent. Given the systemic flaws observed in VCMs affecting interactions and incentivisation between market participants, such as credit inflation and double counting^27,28^, VBMs must integrate lessons learned early to prevent such detrimental dynamics^22,34^. Second, the current low maturity of VBMs allows them to still do so before large-scale market expansion.

We have applied all three components to the VBM context and identified multiple gaps in their fulfilment, which may lead to market failure in VBMs. Table 2 gives an overview of the identified challenges in the current setup of VBMs and potential mitigation measures, both informed by VCM experiences.

**Table 2:** Challenges and mitigation measures in VBMs across components of functioning markets

Component	No.	Challenges	Mitigation measures
Definition of the right —Should be clear	#1	Comparability among BCs of different origins	Division into semi-standardised classes, priced differently
	#2	Uncertainty around BC-related claims	Clarification and advice on the kind and timing of BC-related claims
	#3	Non-like-for-like compensation	Clarification of permitted offsetting scenarios & prohibition of non-like-for-like compensation
Allocation of rights —Should be fair	#4	Exclusion of IPLCs	Benefit-sharing mechanisms
	#5	Uncertainty around ownership	Thorough due diligence and focus on established production systems
Exchange —Should be easy	#6	Credit inflation	Independent VVB appointment, regional panels as auditors and staged payments to align incentives
	#7	Double counting	Establishment of an international oversight body to strengthen transparency, responsible for an independent BC registry, public access to project documentation and the sharing of lessons learned
	#8	Insufficient reputational damage	Introduction of fraud detection agents who drive civil dispute systems to reinforce penalties

The original right over a good or service to be exchanged must be as clear and specified as possible^49^. Applied to BCs, part of this component is already covered at the unit level, where it should be clearly defined what a BC represents, as the findings of the previous section suggest (a certificate about a unit of positive biodiversity outcome fulfilling all five key criteria). In other parts, the definition of the right over BCs remains vague, which lowers the value of the right as uncertainty is involved and might lead to non-participation of actors due to too high transaction costs^49^.

#### Challenge #1: comparability among BCs

While individual BCs can be defined clearly, cross-system comparability remains limited. The BCA^24,34^ calls for semi-standardisation of metrics based on ecosystem type, geography, or species group, forming “classes” of BCs that can be priced differently depending on the cost and difficulty of achieving outcomes. Such classification would even the playing field for those PDs who choose high-integrity metrics and issue BCs ex-post, outcome-based (Interview #3, Impact Director).

#### Challenge #2: uncertainty around BC-related claims

The ability to make a biodiversity-related claim (whether compensatory or contributory) is a key demand driver^12,50^. However, uncertainty around what claims can be made, when, and under which conditions^27,34^ (Interview #3, Impact Director) deters buyer participation as companies do not know what to do with the right they buy, hence, there is no perceived value-add, or even fear of non-compliance with greenwashing regulation if communicating or commercialising the use of BCs^22^ (Interview #1, Sustainability and Biodiversity Specialist and Environment Specialist). Zu Ermgassen et al.^11^ found this same fear of non-compliance among investors within broader biodiversity finance.

It is thus necessary to streamline the kind and timing of claims a buyer can make based on BCs^51^, a requirement supported also by interviewees (e.g. Interview #1, Sustainability & Biodiversity Specialist and Environment Specialist). Regulation on BC-related claims should be specific; linked to BC class and issuance method^22–24,51^. Experience from VCMs shows that regulations such as the proposed EU Green Claims Directive (https://eur-lex.europa.eu/legal-content/EN/TXT/?uri=COM%3A2023%3A0166%3AFIN) have brought more clarity. Some alliances^23,34^, methodology developers (e.g. Interview #3, Impact Director) and BC schemes (e.g. natureplus^52^) draft guidance on BC claims, and anticipate guidelines to develop in parallel with voluntary frameworks (e.g. TNFD and SBTN) and regulations (e.g. the previously mentioned EU Green Claims Directive). But as of now, there is barely any specific national regulation (Interview #8, Climate Advisor). Finland pioneers a BC programme (https://www.environmentenergyleader.com/stories/finland-launches-voluntary-biodiversity-market-to-drive-private-sector-nature-investments,55516) and might serve as a role model for other EU countries in the future. By embedding BCs within a national policy framework, the Finnish approach helps address some of the key barriers identified in this study, such as unclear definitions of rights, lack of standardisation and low demand. A sovereign BC programme provides clearer rules on credit generation and use, institutional support for market participants, and potentially more proactive engagement of private sector buyers. In doing so, it contributes to operationalising core market components, including the clear definition, allocation and exchange of rights, which remain underdeveloped in most VBMs.

#### Challenge #3: non-like-for-like compensation

The definition of the right becomes especially crucial when it comes to the controversial use case of offsetting. Ideally, BCs should come last in the mitigation hierarchy and be used for contribution rather than offsetting^22,26,27^. But the de facto is offsetting one of the most mentioned demand drivers^12^. Considering that biodiversity is highly location-specific^27^, BC offsetting through BCs might be permissible in like-for-like contexts. But it is essential to avoid BCs replacing high-integrity, regulated, like-for-like offsets that “deliver biodiversity gains equivalent in type, amount and duration to the biodiversity losses caused by impacts”^12^. Allowing BCs as “quasi-compensation” without ecological equivalence risks net biodiversity loss^22^. Clear definitions of acceptable equivalence and offsetting principles are therefore essential^27^. In such defined like-for-like contexts, BCs might be used for offsetting, as is practice, e.g. in England with Biodiversity Net Gain^34^. In non-like-for-like contexts, compensation through BCs must be prohibited.

Fair allocation of rights prevents frictions between prior and new users of the right and keeps transaction costs low^49^. This is especially relevant in compliance markets, such as carbon cap-and-trade markets, where rights are established and distributed. But also in voluntary markets, such allocation plays a role when thinking about who previously made use of the grounds now being the basis for BC projects.

#### Challenge #4: exclusion of IPLCs

As shown earlier, the exclusion of IPLCs and other biodiversity stewards has been an issue in VCM and is likely to be repeated in the creation of VBMs. Suggested mitigation measures are to establish fair benefit-sharing mechanisms to balance the interests of previous users and new users of the right (cf. Unit Level).

#### Challenge #5: uncertainty around ownership

In the past, CC projects faced legal issues around liabilities and property rights^30^. To prevent BC projects from similar challenges, Holmlund and Pilstjärna^53^ suggest running a thorough legal due diligence in advance: They prefer to develop and broker BC projects based on production ecosystems like forests, as property rights are typically more clearly established in those landscapes (https://www.youtube.com/watch?v=HDwMR7OkCCg).

Efficiently functioning markets require institutions (formal or informal) that facilitate the exchange of rights to allow for easy transferability and enforcement^49^. Especially in newly emerging markets, institutions can protect market participants from market power by providing information. Further, incentive mechanisms and institutions must prevent post-exchange opportunism by ensuring proper enforcement of rights^49^.

#### Challenge #6: credit inflation

In VCMs, one example of post-exchange opportunism and a major issue has been credit inflation: more CCs were sold than the actual CO_2_ was removed or avoided^26,28^. Issuer-pays structures let PDs pay both the VVB and the standard setter, increasing the likelihood for all parties to benefit from CC inflation^28^: (1) PDs earn paid per credit and may accept less through, but cheaper audits to boost their margin. (2) Standard setters, also paid per credit, prefer projects with high volumes of CCs^22^. They are interested in thorough audits by VVBs to maintain their certification’s reputation, but are faced with VVB undersupply, resulting in delays and/or lower-quality output^28^. (3) VVBs, appointed by PDs, want to keep a good relationship with them for future projects; hence, they might not be as strict in their assessment. CC inflation could have been resolved more effectively if CC buyers had an interest in high-quality CCs, but most lack expertise to differentiate between inflated and non-inflated CCs, typically relying on the standard setters’ reputation^28^, and some might simply not have an interest in investigating whether or not a credit was inflated, and some prefer buying cheaper offsets “so that they can appear sustainable at a lower cost”^28^.

In VBMs, credit inflation risk is generally lower given BCs’ relation to a specific geographical area, but persists in other parts: (1) the possibility to “inflate” credits by choosing metrics that easily show positive change without actually leading to positive biodiversity outcome remains^26^, potentially demanding the same price as higher quality BCs and offering the same opportunity for a buyer to make a claim as higher quality BCs. (2) PDs could choose less thorough methodologies to measure and monitor biodiversity to keep costs low or practice ex-ante issuance of BCs^22^, leaving room for post-exchange opportunism if outcomes are not tied to financial milestones. In this realm, the risk of catering to clients’ preferences for self-selected VVBs also persists. In both cases, the risk for credit inflation can be mitigated if VVBs deliver thorough audits. The incentive to please the individual client can be minimised if VVBs are appointed by standard setters or another independent crediting institution and not engaged by PDs themselves^33,51^. Aide (2024) suggests VVBs to consist of regional panels including biodiversity scientists responsible for project approval, certification and registry, funded by project fees varying with project size and complexity. Whether or not the undersupply of VVBs will be a problem in VBMs as well is not yet foreseeable. Further, staged payments for BCs that are tied to project delivery milestones offer one incentive for PDs to deliver the promised BD outcomes and prevent post-exchange opportunism^30^.

#### Challenge #7: double counting

Market intransparency has led to double-counting in VCMs^28^, meaning that the same credit is sold multiple times. Double-counting is likely to remain an issue in VBMs unless transparency improves. As a form of post-exchange opportunism, it undermines biodiversity goals and prevents markets from functioning properly. Formal or informal institutions can help prevent this: VBMs require transparent, publicly accessible documentation and a central, independent BC registry managed by an international oversight body^12,27,28,49^. This central, standard-setter-independent BC registry would increase transparency on who buys which credit issued by whom, based on which land area, when, and what is done with this credit—similar to what is called for in VCMs^28^. Standard setters would report and update the registry rather than maintaining their own registries. An international oversight body could track issuances, retirements and ownership changes, share lessons learned and maintain overarching guidance for standard setters^12,27^ (Interview #6, Biodiversity Markets Expert).

#### Challenge #8: reputational damage not deterring market participants

In VCMs, scandals over credit inflation and double counting have been made public (e.g. https://www.theguardian.com/environment/2023/jan/18/revealed-forest-carbon-offsets-biggest-provider-worthless-verra-aoe), but reputational damages of standard setters (and VVBs) have not been sufficient to push low-quality CCs or involved market participants (e.g. Verra) out of the market, hurting VCMs’ overall credibility^28,30^. To prevent VBMs from experiencing a similar loss of credibility, market misbehaviour must either be made even more transparent or be penalised more strictly. Proposals for VCMs include fraud detection agents, independent entities rewarded for exposing inflated or duplicate credits, or other fraudulent behaviour, through civil dispute systems and public disclosure of outcomes via accessible platforms^28^. Fraud detection agents’ compensation would be performance-based and financed by governments or corporations with explicit interest in high-integrity markets but without their own capabilities to conduct a thorough due diligence on every credit they buy^28^. Accusations and outcomes of litigation processes would be displayed in an easy-to-understand manner via a publicly accessible platform to increase transparency and reinforce trust in voluntary markets^28^. Civil litigation processes are preferred over formal jurisdictional ones, as honest mistakes can happen due to the high complexity coming with CCs, and a more stringent jurisdiction could turn down voluntary engagement. Such fraud detection agents could similarly increase transparency and trust in VBMs. However, some advocate stricter penalties (exceeding compliance costs) through governmental bodies, e.g. similar to a tax investigation agency, because of the high urgency coming with biodiversity decline and the need to get VBMs right^54^ (Interview #7, Former Policy Advisor).

**Fig. 4:**
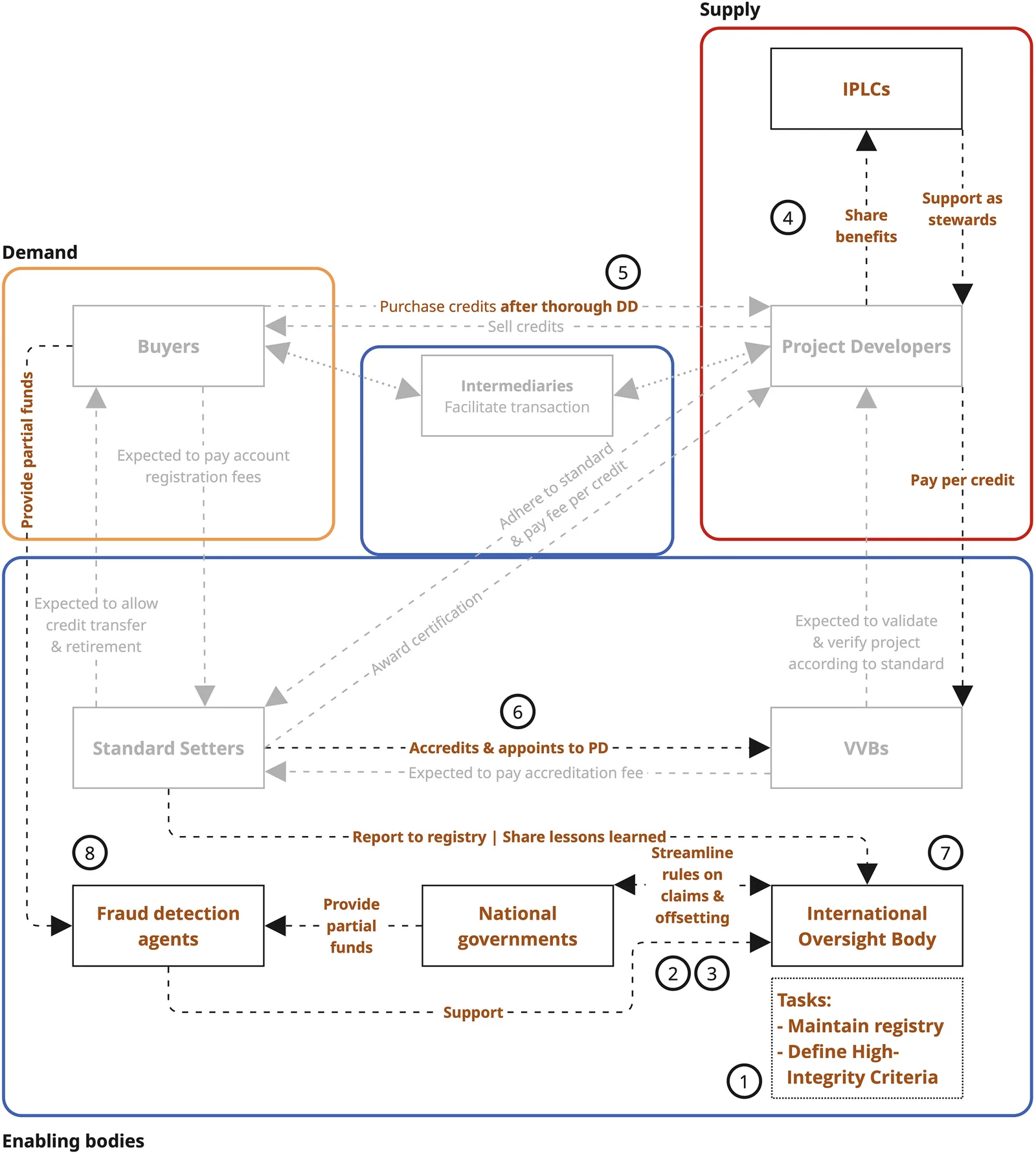
Proposed market architecture for VBMs. Adapted from Fig. 3. The figure presents a proposed enhanced VBM architecture designed to address shortcomings identified in voluntary carbon markets (VCMs). The schematic maps interactions among buyers, project developers (PDs), indigenous peoples and local communities (IPLCs), standard setters, validation and verification bodies (VVBs), governments, registries, oversight institutions and fraud detection agents. Numbered elements correspond to the challenges and mitigation measures described in Table 2 and indicate intervention points intended to strengthen market integrity, transparency and accountability.

The analysis of components that enable functioning markets indicates that while VBMs inherit many vulnerabilities from VCMs, several design improvements can mitigate them. Figure 4. proposes an enhanced market architecture based on the VCM-type architecture (Fig. 3). The numbers in Fig. 4 (e.g. ①) refer to the numbered challenges and mitigation measures listed in Table 2.

## Discussion

Our findings show that BCs are a market-based financial instrument closely related to CCs and early enough in their development to still learn from and avoid carbon market shortcomings. The convergence between our independently derived unit-level criteria and the criteria Zu Ermgassen et al.^35^ and Kim et al.^36^ arrived at through different methods constitutes a form of triangulation that strengthens confidence in both frameworks. Our contribution extends these reviews in three ways. First, our semi-structured interviews with current market participants surface implementation challenges, contested positions and feasibility constraints that are not visible in desk-based reviews, including tensions between scientific integrity and commercial feasibility, divergent views on additionality and early indications of strategic buyer behaviour in an emerging market. Second, while existing reviews concentrate on unit-level integrity, our market-architecture analysis provides a complementary governance framework for how VBMs could be structured to prevent the failures observed in VCMs. Third, we explicitly distinguish environmental integrity criteria from one social integrity criterion, and argue that structural separation matters for how standards, verification and regulation are designed: environmental and social integrity are not only assessed differently, they are also contested on different normative grounds (cf^36^). The criteria at the unit level and the components at the market level all face challenges. While we propose mitigation measures and mechanisms, it is worth discussing their robustness and how it affects the actual positive biodiversity outcome achievable via BCs and VBMs.

Legitimate questions to ask are what the likelihood is that all five key criteria will be met when setting up BC projects, and what the implications are when they are not met.

In our analysis, we have not quantified or categorised this likelihood because most BCs projects are too early in their development to judge the implementation and effectiveness of the proposed mitigation measures^22^. Although recent studies (e.g. ^36^) have assessed BC methodologies and governance frameworks against IAPB criteria, these evaluations focus on alignment with design principles and not observed ecological outcomes over time. Consequently, substantial uncertainty and vagueness remain regarding both the challenges identified and the effectiveness of the proposed mitigation measures.

Many learnings on how to implement the key criteria and mitigate risks around them stem from CCs. But, as pointed out, biodiversity is more complex than carbon. On the carbon side, oftentimes technology-based removal projects have been promoted, partly because additionality is easier to prove compared to nature-based removal or avoidance projects. However, on the biodiversity side, this overreliance on technological solutions when it comes to reversing biodiversity loss is risky. Oftentimes, biodiversity loss is irreversible and related chain reactions are unpredictable, especially in a world facing accelerated climate change^55^.

Furthermore, as other analyses have shown (e.g. https://airtable.com/appXHR0Nau8HqfPa8/shrt5JZqmLMrQgdfF/tblTM31mqfzbGGBtR/viwLkSPMhe3K76baF) most BC projects currently focus on terrestrial rather than marine ecosystems. To evoke holistic positive biodiversity outcomes at the unit level, future project development may need to expand across a wider range of ecosystems, particularly those under greater threat, rather than concentrating on comparatively accessible project types. Related to this, there is a need for education of all parties to ensure the highest scientific rigour and minimise the risk of genuine mistakes in deciding on and setting up BC projects, developing and applying methodology, and validating and verifying BCs. Such upskilling could be facilitated, e.g. through oversight and/or market guiding bodies like the BCA or IAPB, who ideally gather, process and redistribute lessons learned among all VBM participants.

In addition, the results demonstrate that some mitigation measures are proposed for multiple challenges related to the key criteria, e.g. buffers are proposed in relation to durability, additionality, and minimisation of leakage issues, however, they are “only as good as the average quality of the credits they contain”^22^, which is problematic in the case of inherently low-quality BCs, and they can only take up so much of a BC contingent until they are no longer economically viable to the PD. Hence, to ensure a positive biodiversity outcome related to BCs, there is an immediate need for additional risk mitigation strategies. In relation to additionality, the results also highlight one interviewee expressing the philosophy that any intact ecosystem is worth protecting—it is just about the claim that is permitted in relation to the BC bought. While this reasoning circumvents the additionality issue, it is not without challenges: First, focus might be taken away from ecosystems that are actually threatened or already declining and require funding for restoration. Secondly, it opens a discussion on the value-added and how such BCs would be priced compared to others that require more investment and active PD engagement on the ground. As previously mentioned, there are suggestions around price floors, and there could be premiums for “higher value” BCs. It remains to be seen where supply and demand balance out and where price points for different or differentiated BCs crystallise.

The biodiversity outcomes BCs strive for rely on SMART biodiversity targets to define and measure the outcome, and are, thus, more likely to be achieved than outcomes reliant on less SMART targets, as touched upon in the introduction. One interviewed PD revealed that for them, such SMART targets increase the pressure to actually deliver biodiversity outcomes; one reason for that being that the payment can be tied to the target (Interview #2, Fund Director). In their case, buyers would purchase credits on an annual basis, meaning that if targets were not hit, they might stop the business relationship. Such findings provide initial indications that BCs may support biodiversity outcomes in specific circumstances, particularly where financial incentives are aligned with measurable ecological targets. However, evidence from conservation science^56,57^ shows that outcomes vary substantially and depend on factors such as the type of ecological intervention, governance and stakeholder involvement. More proof points would need to be gathered, and direct comparisons would need to be conducted for that to be confirmed. The same PD explained that SMART BCs allow for absolute traceability, which in turn makes storytelling possible (Interview #2, Fund Director). According to the PD, this is one of the main reasons why they engage in VBMs at all, even if the financial gain from BC sales remains marginal. They confirm that nowadays, software allows visualising the actual change and development of the area underpinning a BC project over time. No matter which claim is made by someone buying BCs, the real outcome achieved is traceable in time and location via this platform.

However, whether these potential project-level advantages translate into meaningful biodiversity outcomes at scale ultimately depends on the functioning of the markets in which these credits are embedded. BCs and related market mechanisms are often positioned in policy and practitioner discourse as instruments to mobilise private capital and contribute to closing the biodiversity financing gap^10^, yet evidence suggests that they have so far had a weak track record in mobilising private capital at scale^58^. Moreover, Dempsey^58^ highlights that such “green capital” approaches may persist less because of their effectiveness in addressing biodiversity loss and more because they serve other political and economic functions, e.g. by helping governments and firms signal action and manage legitimacy (overacting on biodiversity loss) despite their limited impact on biodiversity outcomes or working towards solving structural barriers of biodiversity loss.

The results demonstrated that VBMs can function as a market as long as they ensure a clear definition of the rights traded, a fair allocation of those rights, and an easy exchange of rights among market participants. VCMs have faced many shortcomings because they have only partially fulfilled those components, leaving room for credit inflation, double-counting, etc. To ensure VBMs can fulfil their purpose—to help halt and reverse biodiversity loss—a range of incentives and mechanisms need to be altered or put in place, resulting in the proposed improved VBM architecture (cf. Fig. 4).

So far, this architecture is theoretical, partially based on lessons from carbon market experiences, but not yet practice-proven. There certainly remain open questions, e.g. on who exactly will represent the fraud detection agents and whether companies have a high enough interest in high-integrity credits to be willing to pay into a system to increase chances of fraud detection. It also remains questionable whether the public discreditation in cases of fraud detection will reach sufficiently high levels to discipline certain players out of the market. Evidence^59^ from VCMs suggests that persistent demand for low-cost credits can undermine the development of high-integrity standards, as buyers often prioritise inexpensive credits that enable claims of action without requiring substantive changes to underlying activities.

On the other hand, in case stricter monetary penalties were implemented, it is also unpredictable whether market participation would not be negatively affected. Further, it is yet undetermined which institution would form the international oversight body, e.g. whether a new or existing body, or a merger of different bodies (governmental or non-governmental).

Given the current imbalance between supply and demand, clearer rules around permissible claims and credit use will be important for market development. The ability to make claims, including potential offset-related claims, is often discussed as a demand driver^12^. However, interviews and prior research indicate that buyers remain uncertain about what they are purchasing and what claims they may credibly make (unclear definition of their rights)^50^. Such clarification on rules could, e.g. come from the proposed international oversight body in collaboration with national governments. If carefully, like-for-like voluntary offsetting were permitted, companies might be able to make actual “net” claims because they must have calculated and evaluated their biodiversity impacts specifically to be able to compensate according to the like-for-like principle. In all other use cases, it remains difficult to make any “net gain” or “nature positive” claims, as such claims always presuppose the counterbalancing of something negative that has been quantified previously (Interview #1, Sustainability and Biodiversity Specialist and Environment Specialist). Any offset-related use of BCs would need to remain subordinate to the mitigation hierarchy, with avoidance of biodiversity impacts prioritised^26^. Under strict like-for-like requirements, many unique ecosystems or biodiversity hotspots may remain unsuitable for offset-based crediting and would instead require non-compensatory finance.

Historically, hopes were high for VCMs to trigger significant sums of capital for the climate transition; however, up until now, VCMs lag significantly behind the much larger CCMs^28^, having reached only $2 billion/year in 2022 after decades of operation^22^. In comparison, the CCM’s annual trading turnover has reached more than $250 billion by 2021^60^. Current public enthusiasm around VBMs resembles the early days of VCMs^22^. One of the latest market estimates states that since the VBM uptake in 2020 up until September 2024, voluntary BCs worth between $0.325 and $1.870 million have been sold, corresponding to approximately 26.000–125.000 underlying hectares^61^. Even compared to VCMs, these numbers are low. There is no direct comparable “compliance market” as mandatory BOs are usually schemes rather than open markets; still, their market size is estimated to be around $10 billion per year (https://sgradeckas.substack.com/p/biodiversity-markets-reviewing-2024). Hence, market experts (ibid.) and multiple interviewees have suggested moving beyond voluntary commitments and requiring companies to offset any residual harm, also through BCs (e.g. Interview #3, Impact Director). In such cases, BCs would be used in a compensatory manner and thus resemble BOs, highlighting that credits represent the tradable unit, whereas offsetting refers to companies using it in matching biodiversity losses and gains. Such compliance biodiversity markets that would not only allow but demand offsetting might increase the uptake of BCs and mobilise higher amounts of private capital than pure voluntary markets^27^ (Interview #3, Impact Director; #6, Biodiversity Markets Expert). Such markets would need to evolve in combination with companies being increasingly held responsible for their impacts on biodiversity, and offsetting would only be possible if strictly regulated and purchased only in strictly defined like-for-like scenarios to avoid flooding of biodiversity compliance markets with “cheaper” offsets in the form of less stringently controlled BCs.

Some aspirational estimates suggest that combined voluntary and compliance biodiversity markets could reach $162–168 billion in market potential^22^. However, these figures should be treated with significant caution as they represent a theoretical upper-bound estimate rather than a conservative forecast. Such projections often rely on optimistic assumptions regarding corporate uptake and the seamless integration of voluntary and compliance regimes, assumptions that remain largely unproven in the current fragmented market landscape. Around $170 billion would represent a large share of the $200 billion per year that GBF Target 19 calls for in relation to the mobilisation of capital for biodiversity from all sources^6,27^. But in comparison to the $700 billion funding gap for biodiversity pointed out initially, and other initiatives the GBF calls for, e.g. the aspired $500 billion/year reduction in harmful incentives, including subsidies (Target 18)^6^, these capital volumes seem small. Consequently, BCs must be acknowledged as one instrument in a palette of various biodiversity financing schemes.

There are multiple avenues for future research considering the nascency of the field. The ones proposed here either stem from the limitations of this paper, reveal important follow-on research fields, or call for future research to build on and extend the research conducted here.

The primary focus of this paper was the environmental aspects of BCs and the economic facets of VBMs. The social component, particularly concerning IPLCs, is touched upon, but future research could explore associated challenges and mitigation measures around this component in more depth.

In our analysis, we always made a clear distinction between BCs and CCs. However, there is a whole spectrum between the two^23^, which has been excluded in this paper. This spectrum usually covers the concepts of bundling and stacking^61^. Bundling means that multiple ecosystem services are sold together in a single package. This includes, e.g. biodiversity-enhanced CCs, also called CCs with co-benefits. Historically, those credits have traded at a higher premium in carbon markets. Stacking refers to the separate accounting and marketing of distinct ecosystem services derived from the same land^61^. The risk of double-counting is self-evident; however, market experts expect those “shadow biodiversity credit markets” (https://sgradeckas.substack.com/p/biodiversity-markets-reviewing-2024) to exceed standalone BC markets in size. While there are lessons learned from CC projects experimenting with bundling and stacking (cf^22^), future research could further explore this spectrum and analyse synergistic versus cannibalising effects across these products in relation to BCs. A similar market analysis could be conducted with regard to other existing or new biodiversity financing instruments to understand whether, in total, more capital is mobilised for biodiversity than before, and whether the biodiversity funding gap is likely to be closed.

As VBMs mature, the unit-level criteria and market-level components proposed here should be periodically reviewed in light of new empirical evidence, practical experience and regulatory developments. The *BC Requirements Framework* may also be refined for different project types or market contexts.

## Methods

We conducted a qualitative study and chose a mix of methods for the data collection. First, a horizon scan across three research fields was carried out. Second, we led interviews with actual and potential market participants and other experts. After the data analysis, we created the *BC Requirements Framework*, which structures the findings to answer the aim and objectives.

### Data collection

A horizon scan is generally used to generate knowledge about emerging issues and to explore new and uncertain developments^62^. Considering the nascent stage of BCs and VBMs, we chose this method over a systematic literature review based on snowballing as the intention was not to build a complete bibliography on a well-established topic, but to derive an overall understanding of the currently developing BCs and VBMs. Hence, a horizon scan conducted in this paper can be defined as an exploratory review of literature and other media across three research fields: (1) CCs and carbon offsets in CCMs and VCMs respectively, (2) BOs and BCs in compliance markets and VBMs respectively; and (3) other innovative finance schemes mentioned by the GBF and broader approaches for biodiversity finance. The intention of this horizon scan was to understand the status quo and emerging issues and themes (horizons) and lay the foundation to address the research gaps mentioned in the introduction.

Within the exploratory review, priority was given to academic, peer-reviewed articles; however, we also included grey literature such as market reports^24,34,38^, publicly available databases (e.g. https://airtable.com/appXHR0Nau8HqfPa8/shrt5JZqmLMrQgdfF/tblTM31mqfzbGGBtR/viwLkSPMhe3K76baF), newsletters (e.g. https://sgradeckas.substack.com/p/biodiversity-markets-reviewing-2024; https://sgradeckas.substack.com/p/deep-dive-biodiversity-credit-sales) and other media, such as recorded interviews and webinars^39^. These sources were found via the online library Primo (accessed via KTH— Royal Institute of Technology), Google Scholar and the Google search engine. Keywords used for those searches were: biodiversity credit (market), biodiversity banking, biodiversity investment, biodiversity lending, biodiversity offset, carbon market, compliance markets, EU ETS, market theory, nature credit (biodiversity), payments for ecosystem services. Some recently published academic papers were also found via scholars’ posts on the social network platform LinkedIn.

The horizon scan primarily yielded results for the unit level. Therefore, interviews were conducted with actual and potential VBM participants and other experts in climate and biodiversity, with a primary focus on the market level. The role of the interviews was threefold. First, the interviews served as a validation and/or objection to findings from the horizon scan, confirming or challenging some of the theoretical concepts in practice. Second, the interviews filled in where the literature is currently falling short. As mentioned previously, most BC literature concerns the credit itself (unit level), but less so the market. The mix of interviewees was useful in this case to gain a holistic picture of different perspectives. Third, the BC space is moving so quickly that literature which is published now and presents research from a year ago might be outdated already. Therefore, it was insightful to obtain recent information.

In total, eight interviews were conducted from March 3 until April 25, 2025. All interviews took place online (via Zoom), were audio-recorded with permission, and lasted between 28 and 46 min. Recording consent was secured orally from all interviewees. Seven interviews were conducted in English, one interview in German, as both interviewee and interviewer were native speakers. The interviews were semi-structured. Semi-structured interviews are a qualitative data collection method, where an interview guide is prepared, but the flexibility to deviate from this guide during the interview, to ask follow-up questions and to explore additionally arising, relevant topics is granted^63^. We decided to use this method as it allows for a mix between focus and autonomy^63^ and seemed more suitable for interviews on this complex and explorative topic than closed-ended questions.

We developed tailored interview guides for each type of market participant. Core questions focused on the interviewee’s actual or potential role in VBMs, their experiences and perceptions, and their views on other market participants previously identified by the authors or emerging in practice. Interviewees were also asked about shortcomings of VCMs and potential conflicts in participant interactions. Given the diversity of expertise in and knowledge about VBMs, conversations often deviated from the guide, ranging from broad reflections on the climate–biodiversity crisis to technical discussions of biodiversity monitoring tools. Insights from earlier interviews informed subsequent ones, allowing us to confront interviewees with new knowledge or ideas. This approach was not formal hypothesis testing but a flexible “pulse check” of the market. An exemplary interview guide used with a PD (anonymised) is included in the Supplementary Document.

Although semi-structured interviews are typically conducted with one respondent^64^, we allowed for two interviews, including two people, to respect the interviewees’ time. The entire overview of interviewees, including their in-text references, is summarised in Table S1 (Supplementary Document).

This study followed the ethical procedure of KTH Royal Institute of Technology. Further details on how interviews were set up and how consent was obtained can be found in the Supplementary Document.

### Data analysis

For the horizon scan, we categorised findings from papers, reports and other media into the three research fields previously mentioned and collected these within one research document. It became evident that BCs stem from the same school of thought as CCs, but to justify a transfer of learning, we systematically mapped similarities and differences between the two. This pre-analysis is presented in Table S2 (Supplementary Document). and led to the conclusion that despite some differences, they are similar enough so that learnings from the carbon side can indeed be applied to the biodiversity side. It also appeared that there were lessons learned specific to the credit itself (what we defined as unit level) and to the markets where those credits are traded (what we defined as market level). Consequently, we differentiated all types of lessons learned from the carbon space into the unit and market level. The same was done for specific lessons learned from already existing BC projects.

To ease access to information when writing the paper, we used a tagging system both within the citation software Zotero and also within the research document with all findings. Tags included, among others, “unit level,” “market level,” “carbon credit lesson learned,” “demand driver,” “challenge,” “solution,” “risk mitigation measure,” “tech,” “regulation,” and “maturity.” Further, whenever a finding was used for the final paper, it was marked in colour to avoid using the same information repeatedly.

After conducting the horizon scan, the findings for the unit-level part of the paper were largely comprehensive. However, for the market level part, findings from the literature have been limited. This is why we conducted the interviews additionally.

Information obtained through the interviews was processed in two steps. First, we took notes on the most important insights while conducting the interviews. This high-level, summarising information fed into the market level findings already and served as an orientation to build the questionnaire for the next interview in case new questions opened. Second, after all interviews were conducted, we used the software Otter.ai for audio transcription. We translated the interview conducted in German, partially relying on the software deepl.com. The transcriptions allowed us to revisit the interviews and extract highlight quotes. The highlight quotes were collected in a table, sorted by interviewee. Finally, some of the quotes extracted were used directly or indirectly for the paper, primarily within the market-level part.

To present the analysis from horizon scan and interviews clearly and comprehensively, we propose a *BC Requirements Framework* (cf. Fig. 2). The framework is not a methodological approach but represents an analytical tool to organise and communicate the results in a structured manner. At the unit level, the split into key criteria was natural as they largely stem from the BCA definition. All criteria were evaluated through three lenses: theoretical definition, practical challenges and mitigation measures. A lens describes a particular dimension to evaluate something (e.g. a criterion) through and highlight particular points about it^65^. To ground the market-level analysis theoretically, we applied the market theory of Davidson and Weersink ^49^ on key components required for any market to function properly to VBMs. After identifying key market participants and their interactions, we analysed associated challenges and mitigation measures using the same three lenses mentioned above and grouped them under the relevant components.

The interview sample is composed primarily of actors actively engaged in the BC and broader biodiversity finance ecosystem. While this provides valuable perspectives on emerging market dynamics, it also introduces the possibility of constructive or vested bias, as many participants are professionally or financially embedded in the development of these markets. This may lead to more optimistic assessments of market potential and downplaying of structural limitations. The findings should therefore be interpreted as reflecting practitioner perspectives within a rapidly evolving field rather than an externally neutral evaluation. To mitigate this, interview insights were triangulated with findings from the horizon scan, and divergent viewpoints were actively sought across different actor types.

## Supplementary information

Supplementary Document

## Data Availability

The publicly available literature and document sources analysed in the horizon scan are cited in the manuscript and Supplementary Document. No new public repository dataset was generated from the horizon scan. The qualitative interview data generated and analysed during the current study, including audio recordings, full transcripts and interview notes, are not publicly available due to confidentiality and anonymisation commitments made to participants. Given the small and specialised nature of the biodiversity credit and biodiversity finance community, full transcripts and recordings may contain contextual information that could make participants identifiable. The interview guide, participant category information and methodological details needed to interpret the qualitative analysis are provided in the Supplementary Document. Aggregated findings and anonymised evidence from the interviews are reported in the manuscript and Supplementary Document. Additional anonymised derived materials may be made available by the corresponding author upon reasonable request, subject to ethical and confidentiality constraints and provided that disclosure is consistent with the consent obtained from participants.
