# Supplementary material for: A framework for unit-level biodiversity credit integrity and market-level governance
Source: NPJ Biodivers. 2026 Oct 2;5:34. doi: 10.1038/s44185-026-00161-6 (PMC13633254; doi:10.1038/s44185-026-00161-6)
Supplement: Supplementary file 1 [file 44185_2026_161_MOESM1_ESM.pdf]

Supplementary Information on "A Framework for Unit-Level Biodiversity Credit Integrity and Market-Level Governance"

Note: References stated in the Supplementary Document refer to the reference list in the main manuscript.

Overview of Interview Process

Planning and Sampling Strategy

The interviews were planned, conducted, and analysed primarily by L. M. Pelka. Following the horizon scan, an initial set of interview questions was developed and grouped into thematic clusters to guide the inquiry. Interviewees were selected through purposive sampling, with the aim of capturing a diversity of roles across the emerging biodiversity credit (BC) ecosystem, including project developers, standard setters, and financial actors. This approach was chosen to obtain a broad and multi-perspective understanding of market-level dynamics. Potential participants were identified based on their professional involvement in biodiversity finance and related fields, as well as their presumed expertise and relevance to voluntary biodiversity markets.

Participant Outreach and Recruitment

Initial contact with potential interviewees was established via email or the social-professional platform LinkedIn. The outreach communication clearly outlined the research context, institutional affiliation (i.e., KTH, the Stockholm Resilience Centre and the FinBio project), and the purpose of the study, while inviting recipients to participate in a semi-structured interview. Recipients were aware of the voluntary nature of participation. An anonymised example of such an outreach email is provided below:

"Dear [...],

[*Short introduction depending on the author's connection or introduction to that person*]

A short background on myself: I am reaching out as part of the FinBio project hosted by the Stockholm Resilience Centre in collaboration with Stanford and Oxford University and various impact partners. I am currently writing my master's thesis on how to mobilize private capital for biodiversity, particularly through Biodiversity Credits. More specifically, I am looking at what it needs to make biodiversity credits work at unit and at market level and what they can learn from the voluntary carbon market.

I have been following [...] for quite a while already and I would be very curious to learn more about how you approach biodiversity credits, where you see the market evolving, and what mechanisms it needs in place for you to be able to operate successfully.

So far, I have been talking to project developers, standard setters, and financial institutions. I would be really grateful to include your perspective as [...]!

Would you have time for a 30-min zoom call, e.g., [date]?

Thank you so much in advance!

Best,
Lara”

Out of 16 contacted parties, eight parties accepted the invitation to an interview.

Consent, Anonymisation, and Participant Validation

Prior to each interview, oral informed consent was obtained from the interview participants for participation as well as for audio recording. All interviews were conducted via Zoom, recorded with permission, and subsequently transcribed for analysis. According to institutional guidelines at KTH Royal Institute of Technology, no formal ethical approval or additional ethics statement was required for this type of study.

Interview data were analysed in two stages. First, key insights were documented in note form during and immediately after each interview. These high-level summaries informed the ongoing research process, including iterative refinement of interview guides for subsequent interviews. Second, all interviews were transcribed using the software otter.ai; the interview conducted in German was translated into English with partial support from deepl.com. The resulting transcripts enabled a more systematic review of the material, from which salient quotes and recurring themes were extracted. These excerpts were organised in a structured table by interviewee and selectively used, either directly or indirectly, to inform the market-level analysis presented in the paper.

To ensure confidentiality, all interview data were anonymised in the final manuscript. Instead of names or organisational identifiers, interviewees are referenced by interview number and their respective role within the BC ecosystem.

To further enhance transparency and allow participants to retain agency over how their contributions were represented, interviewees were given the opportunity to review the near-final version of the master thesis in which their statements were included in an anonymised form prior to final hand-in and publishing of the thesis via the KTH DIVA-portal. This process was facilitated via follow-up email communication, as exemplified below:

“Hi [...]

This week I defended my master's thesis on biodiversity credits and markets around them. Next week, I will hand in the final version of my thesis which will be published and potentially turned into a paper.

Thank you so much again for your input through our interview - it represented a valuable contribution to my research! Please find the close-to-final version including direct and indirect quotes from you and other interviewees in an anonymised way here: *DocSend Link (no longer active)*

My request: please review Table 1. on page 6 and let me know until latest Thursday (June 5, 2025) at 23:59 whether you would like to remove or change anything about how you are

referred to or about specific quotes throughout the text. Objections I might receive after this deadline can unfortunately no longer be regarded for my thesis hand-in at KTH (for the potential paper based on this, they still can).

Also, please keep this draft confidential and do not yet share it with colleagues. I am happy to send to you the final, handed-in version next week that can be shared. :)

I hope you enjoy reading my thesis and gain valuable insights for yourself from it! Looking forward to your thoughts.

Thank you so much again and best regards,
Lara"

Several interviewees provided feedback following this review process, which led to minor revisions, e.g., in role titles and wording to more accurately reflect their perspectives. Where no objections were raised within the specified timeframe, consent for the use of anonymised material was assumed.

Exemplary Interview Guide (used with Project Developer – anonymised)

<u>Intro</u>

- Can you please tell me more about your role at Company X?

<u>Overview of key questions to set the frame for the interview</u>

- How does your business model work?
- To which degree do you interact with standard setters, VVBs (and other market participants)?
- Is there any role other than that of a project developer that Company X sees themselves in?

<u>BC Business Model</u>

- In your case, can you explain how exactly you define a BC?
- What methodology do you use? Do you base this on specific standard setters?
- How does the sales process of a BC work? What sales channels do you have?
- Have you already sold BCs? For what price? And do you know the buyer's motivation?
- What challenges do you see connected to transboundary sales?
- Are you a for-profit or non-profit organisation? What advantages and disadvantages does each bring to this market?

<u>Unit</u>

- As a seller and supplier in this market: how do you make sure that you provide high-quality credits and how (if so) do you communicate this transparently?
- On this matter: in your opinion, what are the most important lessons learned from VCMs for VBMs?

- How do you work together with Indigenous Peoples and Local Communities? What other environmental safeguards are you embedding?

Market

- How do you work together with VVBs? How do payment mechanisms usually work? What is the frequency of verification?
- Are there any other actors usually involved in a transaction? Are there any marketplaces or brokers yet?
- What are your thoughts on secondary markets?
- On this market level and interplay of actors: in your opinion, what are the most important lessons learned from VCMs for VBMs?

Overview of Interviewees

Table S1. Overview of Interviewees with In-Text References

Categori-sation	VBM partici-pant role<br>*self-declared	Institution	Interviewee's role at institution	Geography	In-text reference
**Demand / Enabling**	Buyer/Broker* (P)	Bank	Sustainability & Biodiversity Specialist<br>Environment Specialist	Sweden	#1
**Supply**	Project Developer* (A)	Conservation Foundation	Fund Director	Sweden, Africa	#2
**Supply / Enabling**	Project Developer* & Methodology Developer* (A)	Biodiversity Investment Firm	Impact Director	Europe, US	#3
**Enabling**	Broker* (P)	Bank	Sustainability Advisor<br>Sustainability Specialist	Sweden	#4
**Enabling**	Broker* (A)	Broker	Project Manager	Germany	#5
**Enabling**	Market intelligence platform* (P)	Biodiversity Finance Consultancy	Biodiversity Markets Expert	Europe	#6
**Other**	Observer (P)	University	Former Policy Advisor	Sweden	#7
**Other**	Regulator (P)	National Environmental Protection Agency	Climate Advisor	Sweden	#8

Note: In the paper, interviewees are referred to according to their in-text reference and their role at the institution they belong to, and/or their actual/potential VBM role. "Actual" (A) refers to the fact that the interviewee or their organisation has already actively participated in emerging VBMs. "Potential" (P) means that they plan to and/or are likely to engage in VBMs.

Table S2. Overview of similarities and differences between BCs and CCs

**Category**	**Similarities**	**Differences**
Aim	Both aim at improving the status quo, addressing the biodiversity and climate crisis respectively.	CCs aim at a *reduction* of greenhouse gas (GHG) emissions compared to a baseline (-), BCs aim at an *increase* in biodiversity (+), and *maintaining* functioning ecosystems (=).
Credit	Both attempt to package biophysical processes/ecosystem services into a measurable, tradeable unit, abstracted enough to be commodified.	CCs represent one ton of Carbon Dioxide (Equivalent) (CO_2(e)) avoided or removed (https://www.unep.org/topics/climate-action/climate-finance/carbon-markets). BCs represent biodiversity, which is more complex to measure because a) there is not a single, sufficiently describing indicator, but a need to combine multiple indicators, and b) the value attributable to biodiversity is not binary[26,27].
Projects	The unit's basis is always a project. Both credits face two underlying project types: avoidance projects (CC: avoided/reduced CO_2 emissions, BC: avoided biodiversity harm) and projects that aim at changing the status quo (CC: removal/sequestration projects, BC: restoration projects)[24,28,29].	For BCs, there is an additional third type of projects: maintenance projects aiming at preserving ecosystems as they are[24].
Compliance markets	There are regulated markets in both the carbon and biodiversity space: CCMs and mandatory biodiversity offsetting schemes, both of which have existed for several decades already (cf. Figure 1.). In both types of markets (limited) offsetting is allowed, even necessary, as regulation demands companies to adhere to emission reduction targets (in case of CCMs), or to remediate environmental harm in building and infrastructure projects (in case of biodiversity offsetting schemes)[27,28,30,31].	CCMs are mature markets allowing for the trading of units within and across national borders (e.g., the EU Emissions Trading System) (https://climate.ec.europa.eu/eu-action/eu-emissions-trading-system-eu-ets/what-eu-ets_en). Mandatory biodiversity offsets represent schemes rather than markets and exist only on a national scale (e.g., Germany, England). CCMs operate under a cap-and-trade-system including the equivalence principle[30], allowing units to be traded as fungible units (meaning they are used for offsetting emissions anywhere along the value chain), while in mandatory biodiversity offsetting schemes there are no cap-and-trade systems and offsetting is limited to like-for-like scenarios. Units traded via CCMs are "carbon credits" (CCs), while the units initiated/purchased in mandatory offsetting schemes are called "offsets" (cf. Figure 1.).
Voluntary markets	CCMs serve as role models for both VCMs and VBMs. Voluntary markets are largely unregulated and buyers, mostly companies, participate to show commitment beyond regulatory requirements[28]. One of the main drivers for buyers to participate in both markets are claims they can make based on this engagement.	VCMs have existed for longer than VBMs. Companies engaging in VCMs usually do so to meet their own "net zero targets" and they purchase units to compensate for any remaining emissions[28,30]. Those units are called "carbon offsets". Offsetting is not allowed in VBMs as biodiversity impacts are not fungible, which is why companies engaging in those markets do so to contribute to biodiversity conservation and restoration efforts[26,27]. The units traded in those markets are called "biodiversity credits" (BCs).
